# Chimeric Antigen Receptor T-cell (CAR-T) Therapy in Autoimmune Diseases: A Narrative Review

**DOI:** 10.7759/cureus.116377

**Published:** 2026-09-16

**Authors:** Irviana A Molina-Garcia, Joselyn G Oliveros-Perez, Stephanie D Molina-Garcia, Anyuli P Guevara-Ostos, Maria F Blanco-Contreras, Ludwig M Camacho-Flores, Luisana S Rodriguez

**Affiliations:** 1 Dentistry, University of the Andes, Mérida, VEN; 2 General Medicine, University of the Andes, Mérida, VEN; 3 Dentistry, Universidad de Carabobo, Valencia, VEN; 4 General Dentistry, Boston University School of Medicine, Boston, USA

**Keywords:** allogeneic car-nk cells, autoimmune diseases, b-cell depletion, car-t cell therapy, immune reset, systemic lupus erythematosus

## Abstract

Autoimmune diseases are chronic immune-mediated disorders that, in their systemic forms, such as systemic lupus erythematosus (SLE), systemic sclerosis (SSc), idiopathic inflammatory myopathies, and antineutrophil cytoplasmic antibody (ANCA)-associated vasculitis (AAV), remain associated with progressive organ damage, impaired quality of life, and excess mortality despite conventional immunosuppression. A substantial proportion of patients experience refractory disease, recurrent flares, and glucocorticoid dependence, underscoring the need for therapies that address the underlying disease process rather than merely suppressing symptoms. Chimeric antigen receptor T-cell (CAR-T) therapy, originally developed for B-cell malignancies, has emerged as a potential paradigm shift by achieving deep, tissue-level B-cell depletion followed by immune reconstitution, a process often described as an "immune reset." This narrative review summarizes the immunological rationale, clinical evidence, safety profile, and future directions of B-cell-directed (anti-CD19) CAR-T therapy in autoimmune disease. Current evidence identifies refractory SLE as the most mature indication, with reproducible drug-free remission, seroconversion, and durable responses across independent cohorts. Early studies in inflammatory myopathies and SSc report encouraging responses and reduced immunosuppressive requirements, whereas evidence in AAV remains preclinical. Toxicities such as cytokine release syndrome and neurotoxicity appear less frequent and less severe than in oncology, although infections and hypogammaglobulinemia require monitoring. High cost, complex manufacturing, and limited long-term data remain major barriers, while allogeneic CAR-natural killer cells and in vivo engineering may improve scalability. Larger controlled studies with longer follow-up are needed before routine clinical use.

## Introduction and background

Autoimmune diseases affect approximately one in 10 individuals worldwide and result from self-antigen-reactive immune system dysfunction that leads to chronic tissue damage [1]. Although they share a mechanism of immune dysregulation, they differ significantly in age of onset, target organ, clinical presentation, and prognosis; most are incurable and require lifelong treatment, and their incidence and prevalence are increasing, with significant socioeconomic and geographic disparities [1,2]. Systemic autoimmune diseases are among the most clinically complex to manage because they frequently involve several organs and present with variable courses, as seen in systemic lupus erythematosus (SLE), rheumatoid arthritis (RA), and multiple sclerosis (MS) [2-4]. These conditions are linked to recurrent disease activity, progressive damage, poor quality of life, and increased mortality; in SLE, mortality remains two to three times higher than in the general population, with infection, cardiovascular disease, and renal involvement as leading causes of death, and the burden on patients and health systems is high owing to long-term treatment, accumulating morbidity, and healthcare expenditure [1,2,5-7].

Although disease control has advanced with therapeutic innovations, treatment still relies on conventional immunosuppressants, corticosteroids, biologic drugs, and targeted therapies. These agents do act on inflammatory and immune mechanisms and reduce organ damage, but they less consistently produce durable, treatment-free remission or restore immune tolerance [7,8]. Despite biologic and targeted treatment, a significant proportion of patients do not achieve or maintain remission or low disease activity and have persistent symptoms, recurrent flares, and glucocorticoid dependence, and long-term therapy may cause immunosuppression, osteoporosis, hypogammaglobulinemia, infection, and other toxic effects [3,9-11]. Cumulative glucocorticoid exposure contributes to irreversible organ damage and to damage accrual [3]. Limited response has given rise to the term difficult-to-treat or refractory autoimmune disease, and there is a critical need for therapies with durable efficacy that can restore immune tolerance and improve long-term outcomes [5,11].

Chimeric antigen receptor T-cell (CAR-T) therapy has emerged as one such strategy [4,11]. It can produce deep B-cell depletion, drug-free remission, and immune reprogramming [4,7,8,11]. The concept of an “immune reset,” which we present as a proposed biological model rather than an established restoration of tolerance, refers to the temporary disappearance of pathogenic B-cell subsets followed by reconstitution of the B-cell compartment primarily with naive or less autoreactive cells; long-term tolerance has not been proven, and the supporting studies remain few, small, and short in follow-up [10]. CAR-T in autoimmune disease therefore remains investigational.

CAR-T was initially designed for hematologic malignancies but, after success in refractory B-cell cancers, has been translated to B-cell-driven autoimmune diseases [7,8,11]. This review focuses primarily on autologous anti-CD19 CAR-T therapy and treats allogeneic CAR-T, CAR-natural killer (NK), dual-target and B-cell maturation antigen (BCMA)-directed constructs, and in vivo engineering as distinct emerging platforms with separate and generally weaker evidence. It addresses the following question: in adults with severe autoimmune disease resistant to conventional immunosuppression, what is the current evidence for the efficacy, safety, and clinical application of anti-CD19 CAR-T-cell therapy, and what are the main barriers to its use in routine practice?

## Review

Search strategy

This is a structured narrative review conducted in accordance with the Scale for the Assessment of Narrative Review Articles principles rather than as a systematic review [12]. We searched PubMed/MEDLINE, Embase, and Scopus for records published between January 2020 and July 2026, using combinations of the terms “CAR-T”, “chimeric antigen receptor”, “autoimmune diseases”, “systemic lupus erythematosus”, “lupus nephritis”, “systemic sclerosis”, “idiopathic inflammatory myopathies”, “ANCA-associated vasculitis”, “CD19”, “BCMA”, “CAR-NK”, and “immune reset”, with the final search performed in July 2026. Original clinical studies, prospective and retrospective observational studies, case reports and series, clinical trials, systematic reviews, and high-quality narrative reviews published in English were considered; studies addressing exclusively oncologic applications were included only where they provided mechanistic or historical context, and additional records were identified by manual screening of reference lists. Records were screened by title and abstract and assessed in full text by the authors, with disagreements resolved by discussion. Consistent with the narrative design, no formal risk-of-bias appraisal, PRISMA flow diagram, or quantitative synthesis was undertaken, and where independent cohorts may share patients, this is noted. The review focuses on systemic, B-cell-mediated autoimmune diseases in adults, with pediatric evidence noted where available; B-cell-depleting agents such as rituximab and bispecific antibodies are cited only as background.

Immunological rationale and mechanism of action

B-cell involvement in disrupting immune tolerance underpins the therapeutic use of CAR-T cells in autoimmune diseases. B cells produce pathogenic autoantibodies and participate in antigen presentation, cytokine secretion, and activation of autoreactive T cells, all of which can contribute to chronic inflammation [13,14]. While classic B-cell-targeted therapy has improved disease control, it has not completely eradicated the pathogenic B-cell population, which hinders the establishment of long-term immune tolerance [15]. CAR-T therapy has thus been developed to achieve deep B-cell depletion and immune reconstitution, the biological foundation for an immune reset [13,14].

CAR-T cells are T lymphocytes genetically engineered to recognize antigen in the absence of major histocompatibility complex (MHC) presentation [15]. The engineered receptor consists of an extracellular single-chain variable fragment, a hinge region, a transmembrane domain, and intracellular signaling domains [15]. Antigen recognition activates the CD3ζ signaling domain, and co-stimulatory molecules such as CD28 or 4-1BB promote proliferation, cytokine production, persistence, and survival, markedly enhancing the potency of second-generation CAR-T products; MHC-independent recognition activates T cells rapidly, expands the clone, and generates memory T cells that can continue immune surveillance [16,17].

CD19 has been identified as a common target across autoimmune diseases because it is expressed at most stages of B-cell maturation, allowing elimination of precursor, mature, and plasmablast B cells [15]. Unlike anti-CD20 monoclonal antibodies, which are less effective at eradicating multiple pathogenic B-cell subsets, this broad targeting is a distinct benefit of CAR-T therapy; however, surviving plasma cells often lack CD19 and may continue to produce pathogenic autoantibodies [13]. Dual CD19/BCMA targeting has therefore been developed to more completely deplete the plasma cells responsible for ongoing humoral autoimmunity [14,15]. Unlike monoclonal antibodies, CAR-T cells can infiltrate inflammatory sites, proliferate after antigen recognition, and kill autoreactive B cells without relying on antibody-dependent cellular cytotoxicity or complement activation, enabling more complete reduction of autoreactive B-cell reservoirs in lymphoid organs and inflamed tissues not reached by routine treatments [14,18].

Immune reconstitution and the notion of immune reset are built on profound B-cell depletion [14]. During hematopoietic recovery, the compartment is dominated by naive and transitional B cells, whereas pathogenic memory B cells remain significantly depleted [14,15]. This remodeling has been associated with a return toward immune tolerance, elimination of pathogenic autoantibodies, normalization of immunological biomarkers, and recovery of immune networks, and the degree of reconstitution appears to depend on the extent of depletion, with more comprehensive depletion favoring a more lasting balance rather than transient immunosuppression [13,14].

Lymphodepleting chemotherapy before infusion (fludarabine plus cyclophosphamide) allows CAR-T cells to expand and persist by reducing endogenous lymphocyte competition and increasing homeostatic cytokines [13,14]. After antigen recognition, CAR-T cells proliferate and may survive as long-lived memory cells that provide continued surveillance and B-cell depletion; the optimal duration of persistence remains under investigation, but longer persistence may help keep autoreactive B cells in check [13,16,17]. In summary, CAR-T deeply eliminates autoreactive B-cell populations and is followed by immune reconstitution toward a more tolerant state, providing the biological rationale for the clinical evidence reviewed below [13,14].

Clinical evidence in SLE

The first clinical evidence that CAR-based immune effector cell therapy can induce long-term remission in an autoimmune disease came from a patient treated for severe, treatment-refractory SLE, reported in the primary study by Müller et al. and summarized in subsequent reviews [19]. Since that initial report, SLE has accumulated the most clinical experience with this strategy, and several prospective cohorts have evaluated different CAR designs and manufacturing methods in carefully selected patients for whom conventional therapy had failed [7,20-23].

The index patient was a 20-year-old woman with lupus nephritis and joint, serosal, cutaneous, and cardiac-valve (Libman-Sacks) involvement that had not responded to multiple immunosuppressive and B-cell-targeted agents; her disease resolved clinically and serologically within about five weeks of receiving autologous CD19-directed CAR-T cells [19]. Five further patients treated in the same primary series achieved drug-free remission within three months by Definition of Remission in Systemic Lupus Erythematosus (DORIS) criteria, with declining peripheral B cells and significant clinical improvement, and the return of peripheral B cells suggested genuine restoration of tolerance rather than transient suppression [19]. A subsequent report of 15 patients with multiple autoimmune diseases (eight with SLE) confirmed sustained drug-free remission and normalized Systemic Lupus Erythematosus Disease Activity Index (SLEDAI) scores over a median follow-up of nearly 12 months [24]; similar results have since been reported at other centers using autologous, allogeneic, dual-target, and CAR-NK strategies, which should not be regarded as carrying equivalent levels of evidence [7,21,23].

Laboratory findings closely tracked clinical improvement: disease-associated autoantibodies such as anti-double-stranded DNA and anti-Sm decreased or disappeared, complement normalized, proteinuria fell, and SLEDAI-2K frequently reached and remained at zero, with many patients withdrawing entirely from other immunosuppressants and glucocorticoids [9,11,19,20]. Follow-up reports describe remission ranging from a few months to more than two years, even after B-cell numbers recover, with predominantly naive returning B cells and rare relapse, suggesting that the clinical advantage outlasts the period of total B-cell depletion [11,19,20,22,23]. Treated patients have generally had severe multiorgan disease, particularly lupus nephritis, with prior failure of cyclophosphamide, mycophenolate mofetil, calcineurin inhibitors, rituximab, belimumab, and glucocorticoids [20-23]. Reproducibility across independently managed cohorts has made refractory SLE the principal setting for evaluating CAR-based cellular therapy in autoimmunity and has prompted extension to other severe diseases [7,21,23]. Reported cohorts and their key features are summarized in Table 1, which also flags where publications may overlap.

**Table 1:** Representative reported cohorts of CAR-based cellular therapy in autoimmune disease, by platform. Levels of evidence differ substantially across platforms, and some cohorts may share patients CRS: cytokine release syndrome; ICANS: immune effector cell-associated neurotoxicity syndrome; SSc: systemic sclerosis; SLE: systemic lupus erythematosus; ACR: American College of Rheumatology; NK: natural killer; CAR-NK: chimeric antigen receptor-natural killer; DORIS: Definition Of Remission in Systemic Lupus Erythematosus

Cohort (reference)	Disease	n	Platform	Follow-up	Key efficacy/remission	Safety notes
Müller et al. [19]	SLE (index + 5)	6	Autologous anti-CD19	Up to ~24 months	DORIS drug-free remission in all by 3 months	Mostly grade 1 CRS; no severe ICANS
Wong et al. [23]	Mixed autoimmune	Pooled	Mixed (mostly autologous)	Varies	Pooled remission and seroconversion; may include overlapping patients	Low-grade CRS predominant
Müller et al. [25]	Myositis, SSc, SLE	Varies	Autologous anti-CD19	Up to 6 months	Major/moderate ACR response; no SSc progression at 6 months	Manageable CRS
Auth et al. [26]	Diffuse SSc	6	Autologous anti-CD19	Median 16 months	~1/3 fall in skin score; no immunosuppression restart	Low-grade CRS
Wang et al. [27]	Myositis, SSc	Few	Allogeneic anti-CD19 (donor)	Limited	Deep remission in refractory necrotizing myopathy	Limited data
Gao et al. [28]	Refractory SLE	18	Allogeneic CAR-NK	9 with >12 months	6/9 DORIS remission at >12 months	1/18 grade 1 CRS; no neurotoxicity
Zeng et al. [29]	Refractory SLE	5	Cord blood CAR-NK	Limited	Remission/low disease activity in a subset	Favorable early safety

Beyond lupus: myositis, SSc, AAV, and emerging indications

While SLE is the initial disease model, evidence is expanding to inflammatory myopathies, systemic sclerosis (SSc), antineutrophil cytoplasmic antibody (ANCA)-associated vasculitis (AAV), and other conditions, with less robust data than in SLE [24,30]. CD19-directed CAR-T has been studied in antisynthetase syndrome, dermatomyositis, and immune-mediated necrotizing myopathy, with reports of normalized creatine kinase, improved muscle strength, cleared pulmonary inflammation, and reduced immunosuppression [30]. In an early-phase cohort of 15 patients, the three with idiopathic inflammatory myopathy achieved a major or moderate American College of Rheumatology response and were steroid-free [24], and four of five myositis patients in the CASTLE basket trial achieved a major or moderate response and were steroid-free [25]. Deep remission has also been reported in a single patient with refractory necrotizing myopathy treated with allogeneic donor-derived CAR-T cells [27], although such allogeneic experience is limited to a few cases [31].

The most compelling non-lupus evidence is in SSc. In six patients with diffuse cutaneous disease, skin-thickening scores improved by about one-third at three months, digital ulcers decreased, and hand function improved, with none restarting immunosuppression at a median follow-up of 16 months; radiographic gains reflected reduced ground-glass change rather than regression of established fibrosis, suggesting stabilization [26]. At six months, nine SSc patients in the CASTLE cohort showed no progression [25]. Evidence for AAV is promising but earlier: in a murine myeloperoxidase (MPO)-ANCA model, CD19 CAR-T cells localized to the kidneys, reduced autoantibody-producing cells, and prevented glomerular injury [32]; a broader synthesis suggested disease control without immunosuppression or life-threatening effects [33]; and although MPO- and PR3-reactive B cells together with T-cell- and monocyte-mediated injury provide a biological rationale for CD19 targeting, published human experience is limited to a few short-term, uncontrolled cases with favorable renal outcomes and glucocorticoid tapering [34].

The field continues to broaden. An international working group has proposed a framework for evaluating CAR-T in pediatric-onset rheumatic disease, with age-adapted eligibility, dosing, and monitoring [35]. Among 10 patients with RA treated with B-cell-directed therapy, nine with seropositive disease achieved durable drug-free disease, while the single seronegative patient relapsed after an initial response [36]. Other emerging indications include Sjögren's disease [33], neurological autoimmune disorders such as MS and myasthenia gravis [10], and pemphigus vulgaris, so far only in preclinical models [31]. Collectively, these studies show persistent clinical improvement, reduced steroid use, and continued medication-free control [24,25,36], although comparisons are limited by differing disease definitions, outcome measures, and follow-up, and larger controlled trials are needed to establish durability and identify who benefits [25,31,33].

Safety, toxicity, and practical considerations

Safety data specific to autoimmune cohorts are still limited but generally reassuring: in the SLE and myositis/SSc series reported to date, cytokine release syndrome (CRS) has been predominantly low grade and immune effector cell-associated neurotoxicity syndrome (ICANS) infrequent, and CAR-NK series have reported very low CRS rates and no neurotoxicity [19,25,26]. Much of the detailed toxicity taxonomy, however, derives from oncology populations, where disease biology, tumor burden, and prior therapy differ; these data provide indirect context and cannot be extrapolated directly to autoimmune disease, in which lower antigen burden may underlie the milder observed toxicity.

With that caveat, the oncology literature frames the toxicities to monitor. A semimechanistic cellular kinetic model of 217 patients across seven trials characterized CAR-T cells as a “living drug” undergoing distribution, proliferative expansion, contraction, and long-term persistence, with proliferation and reduced contraction correlating with response better than infused dose [37]. The most prominent acute toxicities are CRS and ICANS, managed mainly with tocilizumab and corticosteroids; others include immune effector cell-associated hematotoxicity, delayed movement disorders with anti-BCMA constructs, and on-target off-tumor injury with GPRC5D- and mesothelin-directed products [38]. In a registry of 1,916 patients with large B-cell lymphoma, CRS and ICANS were common and often co-occurring; more severe CRS predicted worse outcomes, and although severe CRS declined over time with earlier tocilizumab use, severe ICANS did not, underscoring the difficulty of neurotoxicity control [39]. The immune effector cell-associated HLH-like syndrome has been defined by ASTCT criteria (rising ferritin, transaminitis, coagulopathy, cytopenias) with a stepwise treatment approach [40].

Longer-term immune complications require monitoring. By analogy with rituximab-treated populations, hypogammaglobulinemia and late-onset neutropenia may predispose to respiratory, viral, and opportunistic infection, so follow-up increasingly includes serial immunoglobulin measurement, functional antibody testing, infection surveillance, and immunoglobulin replacement [41,42]. Experience translating oncology CAR-T into routine practice, drawn from 180 patients treated with ciltacabtagene autoleucel for myeloma, indicates that outcomes can be maintained in older, less selected patients through multidisciplinary toxicity management, bridging therapy, and long-term surveillance for secondary myeloid neoplasms, infection, and hypogammaglobulinemia; its direct relevance to autoimmune practice is limited but instructive [43]. For autoimmune applications specifically, a multistakeholder working group has recommended disease-activity-based eligibility, reconsideration of conditioning to preserve fertility, modified dose-limiting-toxicity frameworks, infrastructure for long-term safety monitoring in SLE, and surveillance for clonal hematopoiesis as an emerging secondary-malignancy risk [22]. An effective safety program must therefore integrate cellular kinetics, acute and delayed toxicities, immune reconstitution, infection prevention, and disease-specific monitoring.

Challenges and future directions

Toxicity is not the only obstacle to wider use. Adoption is limited by high cost, complex manufacturing, the need for specialized facilities, and limited evidence of durable benefit [44,45]. Autologous CAR-T infusion costs approximately USD 500,000, which must be read against the substantial lifetime burden of severe SLE, including hospitalizations, chronic medication, disability, and complications such as lupus nephritis; an economic model estimated that cumulative disease costs equal the cost of CAR-T after seven to eight years in very severe disease, 11 years in highly active disease, and 20 years in moderate disease, with no meaningful break-even point in mild or inactive disease [46]. These estimates assume durable single-infusion remission without major complications or retreatment and should be interpreted cautiously given the unknown duration of remission. Hospital-based (point-of-care) manufacturing, allogeneic products, and RNA-based approaches have been proposed to simplify and reduce the cost of production [44,46].

Access also depends on reimbursement, infrastructure, manufacturing capacity, and trained personnel. A European analysis of oncologic indications found substantial disparities in national reimbursement decisions and time to access, indicating that reimbursement alone does not ensure access; further delays arise from referral to authorized centers, administrative approval, cell production, and, for autoimmune indications, restricted access to specialized centers [47].

Allogeneic CAR-NK cells are distinct from CAR-T but share the CD19 target and the aim of deep B-cell depletion, and are intended to address limitations of personalized CAR-T. Off-the-shelf products manufactured in advance may offer faster availability, greater scalability, potentially better safety, and lower cost, though these remain theoretical advantages not yet confirmed by direct comparison [28,29]. The two refractory-SLE CAR-NK studies were both small, single-arm trials from China: a series of 18 patients in which nine had more than one year of follow-up and six achieved remission or low disease activity [28], and a second study of only five patients [29]. This concentration in a single country reflects a broader pattern in which clinical development remains focused on a few countries, diseases, and target classes, largely at early stages, so these results should be considered preliminary.

A further approach is to generate CAR immune cells within the patient. In vivo engineering can bypass several steps of standard ex vivo production by delivering genetic material to circulating or resident immune cells [5,44,48], with the main challenges being delivery precision, nonspecific immune activation, nanocarrier immunogenicity, repeat dosing, and control of CAR expression [48]. A murine study generated Epstein-Barr virus nuclear antigen 1-specific CAR-T cells in situ and selectively targeted pathogenic B cells in a model of experimental MS; while application to MS is beyond this review's scope, it offers proof of concept for future autoimmune use and remains preclinical [49].

Limitations

Several limitations temper the conclusions of this review. The clinical evidence is dominated by small, uncontrolled, single-arm cohorts with short follow-up, no comparison against standard of care, and heterogeneous outcome definitions, which precludes pooled estimation and makes informal aggregation of remission or toxicity rates unreliable, particularly where independent publications may share patients. The strongest evidence is confined to autologous anti-CD19 CAR-T in refractory SLE; data for allogeneic CAR-T, CAR-NK, dual-target, BCMA-directed, and in vivo platforms are earlier and should not be treated as equivalent. Much of the safety taxonomy is extrapolated from oncology, and several supporting statements rest on secondary reviews or on indirect (rituximab or myeloma) evidence rather than autoimmune-specific primary data. As a structured narrative rather than a systematic review, this work did not apply a formal risk-of-bias assessment or certainty-of-evidence grading, and publication and reporting biases toward positive early results are likely. These constraints should be weighed when interpreting the encouraging but preliminary signals described here.

## Conclusions

CAR-T therapy holds promise for changing the care of severe refractory autoimmune diseases, but the available evidence is not yet sufficient to support routine use. Published clinical experience remains small and concentrated in a few conditions; most reports are uncontrolled, are not compared with standard therapy, lack standardized outcome measures, and provide limited long-term follow-up. For now, CAR-T therapy should be reserved for carefully selected patients treated in specialist centers or within clinical trials. Larger controlled studies, longer observation, clearer selection criteria, and more scalable, accessible platforms will define its ultimate role.
